# Method of Removal of Aquatic Pond Weed Variably Impacts Presence of Potentially Toxic Cyanobacteria

**DOI:** 10.17912/micropub.biology.002184

**Published:** 2026-06-05

**Authors:** Sarah D Princiotta, Emily Ostergaard, Elizabeth Carroll

**Affiliations:** 1 Biology, Penn State Berks, Reading, PA, US; 2 Biology, Penn State Schuylkill, Schuylkill Haven, PA, US; 3 Biology, Holy Family University, Philadelphia, PA, US

## Abstract

Lakes often exist in two alternative stable states: clear waters dominated by submerged aquatic vegetation and turbid states with an abundance of phytoplankton. Management decisions must balance the value of primary producers with recreational use. We monitored the phytoplankton community surrounding removal of invasive *Potamogeton crispus* , a submerged aquatic perennial. Focus was given to presence of cyanobacteria, which may thrive in the absence of macrophytes by virtue of phosphorus that would otherwise be stored in plant biomass. Results demonstrate that macrophyte removal can lead to an increase in cyanobacteria dominance, with sustained impacts after hand removal.

**Figure 1. Percentage of the phytoplankton community composed of cyanobacteria based on month and form of aquatic plant removal:** Percentage of the phytoplankton community composed of cyanobacteria in aquatic sites where *Potamogeton crispus* was removed by hand or mechanically and otherwise left undisturbed (control). The month of June represents pre-treatment conditions. Individual one-way ANOVA tests were conducted within each treatment site to investigate the influence of removal method over time on the percentage of the phytoplankton community composed of cyanobacteria. Letter cases in the figure indicate results of the post-hoc Tukey HSD.

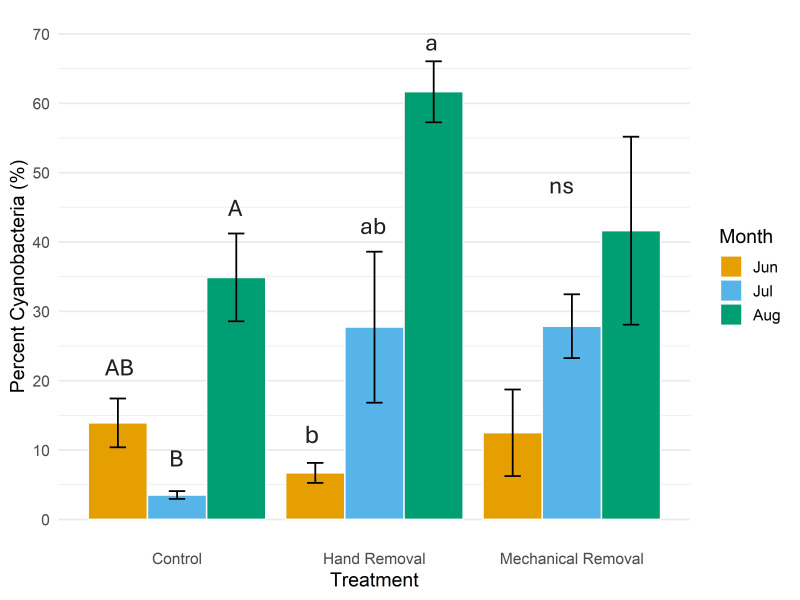



## Description

Despite their evolutionary origins as key aquatic primary producers, dense proliferations, or “blooms” of potentially harmful cyanobacteria are an issue for water quality management in lakes and reservoirs worldwide. The drivers of cyanobacteria dominance and production of toxic secondary metabolites are complex and likely related to environmental conditions associated with global climate change (Paerl et al., 2016). An increased frequency and severity of cyanobacteria blooms are often associated with high surface water temperatures and anthropogenic eutrophication (Kosten et al., 2012; Chapra et al., 2017) Lake managers are tasked with preventing and responding to cyanobacteria blooms that have been implicated in public health consequences for wildlife, pets, and humans (Backer et al., 2013; Trevino-Garrison et al., 2015; Skafi et al., 2021). 

Another threat to freshwater ecosystems is the overgrowth of invasive, submerged aquatic vegetation (Kuiper et al., 2017), which may compromise use of recreational lakes for boating and fishing. Although important to the structure of lake ecosystems as a source of habitat, sediment stability, and nutrient storage (van Donk and van de Bund, 2002; Kovalenko et al., 2010), excess growth of submerged aquatic macrophytes can have negative impacts on water quality when decomposition releases large amounts of phosphorus and consumes dissolved oxygen (Asaeda et al., 2000). There are several methods to manage aquatic macrophytes including removal by either hand or motorized harvesters and chemical treatment with herbicides. Removal of excessive amounts of aquatic macrophytes should be conducted with great caution, as they stabilize the clear water state of shallow lakes (Scheffer et al., 1993). However, it can also be important to balance lake ecosystem health with recreational user preferences.

Aquatic macrophytes may act as natural control agents for cyanobacteria blooms via release of allelopathic compounds (Jasser, 1995), shading, and nutrient competition (Zhou et al., 2017). Field studies that document changes in phytoplankton community structure after removal of aquatic vegetation by various methods are lacking and produce mixed results (Morris et al., 2006; Maredová et al., 2021), especially in lakes that receive a temporally restricted, rather than continuous, removal of dense stands of aquatic vegetation. We took advantage of lake management action to study the impacts of aquatic plant removal ( *Potamogeton crispus)* by either hand or mechanical harvest on the presence of cyanobacteria. A control was provided by a site in which aquatic plants were undisturbed and present at a low density. We hypothesized that removal of *P. crispus* , either by hand or mechanical removal, would lead to an increase in cyanobacteria as representatives in the phytoplankton community, with potentially greater impacts on the hand removal site where fragmented plant matter could act as an inadvertent nutrient source. Removal of *P. crispus* may also enhance cyanobacteria growth due to reduced nutrient competition.

Prior to removal of *P. crispus* , there were no differences in the percentage of the phytoplankton community composed of cyanobacteria across sites (11% ± 2 SE). However, prior to any initiation of treatment, there were notable differences in the abundance of cyanobacteria by site (F _2,3_ = 15.41, p = 0.03), with a higher abundance of cyanobacteria in the site designated for mechanical removal relative to that designated for hand removal. Additionally, the abundance of cyanobacteria at the experimental sites destined for plant removal were not significantly different from the control site.

There was a significant effect of month on the percentage of the community composed of cyanobacteria at both the hand-removal (F _2,4_ = 24.85, p = 0.0055) and control site (F _2,4_ = 25.09, p = 0.0054). At the hand-removal site, the percentage of the phytoplankton community composed of cyanobacteria increased over time from 7% to 62%, with significant differences between June, prior to plant removal, and August (p = 0.0048). There was not a significant effect of month on the abundance of cyanobacteria at the hand removal site, suggesting the observed increase in percentage of the phytoplankton community composed of cyanobacteria may result from changes in community structure. It is possible that non-cyanobacteria members of the phytoplankton community did not respond as favorably to conditions associated with hand removal of *P. crispus* relative to cyanobacteria. Hand removal of *P. crispus* may have increased the availability of photosynthetic active radiation (PAR) and nutrients available to the phytoplankton, of which cyanobacteria are well-adapted at capitalizing.

In the control site where aquatic plants were undisturbed, there was an initial, although non-significant, decline in the percentage of the community composed of cyanobacteria between pre-treatment conditions in June and July. However, cyanobacteria dominance (%) increased significantly between July and August (p = 0.0047). When considering cyanobacteria abundance at the control site, there was a significant effect of month (F _2,4_ = 41.75, p = 0.002), where cyanobacteria abundance also increased from July to August (p = 0.002), and the abundance in August was higher than the pre-treatment conditions (p = 0.01). It is worth noting that whereas the cyanobacteria population was kept low between June and July in the control site, there was a positive response of month on non-cyanobacteria members of the phytoplankton community within the same time frame. These results are indicative of the potentially negative allelopathic interactions between *P. crispus* and cyanobacteria or at least a differential response between the phytoplankton community to the presence of *P. crispus* .

In July but not August, there was a significant effect of site on the percentage of the community composed of cyanobacteria (F _2,3_ = 13.27, p = 0.03). A higher percentage of cyanobacteria were observed in the hand and mechanical removal sites relative to the control site where aquatic plants were undisturbed. Approximately one month after treatment, cross-site differences in cyanobacteria dominance (%) were no longer apparent. It may be interpreted that presence of excessive *P. crispus* had a negative impact on cyanobacteria that was temporarily relieved after removal of the aquatic macrophyte by either hand or mechanical measures. Our methodological approach does not allow us to interpret if this is due to regrowth of *P. crispus* , decomposition of fragmented plant material, or natural differences in cyanobacteria dominance that may occur within a single ecosystem. We suggest future studies to follow impacts of *P. crispus* removal through the fall germination period. This work provides a basis for targeted investigations that seek to balance invasive plant management with ecological health.

## Methods

*Field collection and Enumeration of Cyanobacteria by Microscopy*

The study lake (Z _max_ 14 m, ~24 ha.) is a natural lake of glacial origin located in Pike County, Pennsylvania. Identifying information, including lake name, are not included here per sampling agreement. The vegetation within the drainage basin is primarily forested and the lake has no inlet streams. Routine lake monitoring from a central location on the study lake report a range of summer Secchi depth from 2.5 – 3 m, epilimnetic chlorophyll from 0.42 – 3.4 mg L ^-1^ , and epilimnetic total phosphorus from 4.45 – 5.75 mg L ^-1^ . The phytoplankton community is dominated by cyanobacteria genera that can produce cyanotoxins ( *Limnothrix* , *Aphanizomenon* , *Dolichospermum* ).

Curly-leaf Pond Weed ( *Potamogeton crispus* ), a submerged perennial, was first identified in the study lake during 2020-2021, composing ~12 acres of the shoreline in 2023. In July 2024, a combination of hand removal and mechanical harvest removed ~95% of *P. crispus* from the shoreline. Seven acres were treated by mechanical harvest and five acres by hand removal over a two-day period. The mechanical harvester was able to both cut and collect aquatic macrophytes up to 1.3 m below the surface. Hand removal efforts sought to remove all *P. crispus* , including the roots, but the macrophyte commonly broke mid-stem. A central location within each site, including an area that was not treated (control), was sampled three times over the course of the study period: one week prior to plant removal (June), and 29 (July) and 56 (August) days after removal.

During each sampling event, triplicate sub-surface (0.5 m) water was collected by Van Dorn bottle along a transect, passed through a 153-mm mesh, and stored in 250-mL polycarbonate bottles. Each sample was fixed with a final concentration of 2% Acid Lugol’s and stored in darkness prior to analysis. Samples were concentrated by settling over a 48-hour period after which the remaining ~20 mL was kept for microscopy. A minimum of 300 natural units were identified by light microscopy using a Zeiss Axiovert A1 at 400X magnification using the Utermol method (Paxinos and Mitchell, 2000). Phytoplankton, including cyanobacteria, were identified to genus apart from cells < 5mm diameter that lacked distinctive features, which were grouped as “unidentified nanoplankton.” 

*Statistical Analysis*

All data analysis was conducted in JMP 18.2.2 and the figure was made using R version 4.2.3. Normality and homogeneity of variance were confirmed with a Shapiro Wilk and Levene test, respectively. To meet the assumptions for statistical testing, cyanobacteria abundance and percentage of the community composed of cyanobacteria were log and logit transformed, respectively. To investigate the influence of removal method over time within each site (hand removal, mechanical removal, no removal) a one-way ANOVA with post-hoc Tukey HSD each for cyanobacteria abundance and percentage of the phytoplankton community composed of cyanobacteria. To compare the influence of removal method (hand removal, mechanical removal, no removal) on the percentage of the phytoplankton community composed of cyanobacteria, a one-way ANOVA with post-hoc Tukey HSD was applied for each month after treatment was applied (July, August) with site as the independent variable. Due to significant differences across sites on the abundance of cyanobacteria measured prior to removal method, we did not pursue abundance as a dependent variable for an exploration of the effect of removal method across sites.

## References

[R1] Asaeda Takashi, Trung Vu Kien, Manatunge Jagath (2000). Modeling the effects of macrophyte growth and decomposition on the nutrient budget in Shallow Lakes. Aquatic Botany.

[R2] Backer Lorraine, Landsberg Jan, Miller Melissa, Keel Kevin, Taylor Tegwin (2013). Canine Cyanotoxin Poisonings in the United States (1920s–2012): Review of Suspected and Confirmed Cases from Three Data Sources. Toxins.

[R3] Chapra Steven C., Boehlert Brent, Fant Charles, Bierman Victor J., Henderson Jim, Mills David, Mas Diane M. L., Rennels Lisa, Jantarasami Lesley, Martinich Jeremy, Strzepek Kenneth M., Paerl Hans W. (2017). Climate Change Impacts on Harmful Algal Blooms in U.S. Freshwaters: A Screening-Level Assessment. Environmental Science & Technology.

[R4] van Donk Ellen, van de Bund Wouter J. (2002). Impact of submerged macrophytes including charophytes on phyto- and zooplankton communities: allelopathy versus other mechanisms. Aquatic Botany.

[R5] Jasser Iwona (1995). The influence of macrophytes on a phytoplankton community in experimental conditions. Hydrobiologia.

[R6] Kosten Sarian, Huszar Vera L. M., Bécares Eloy, Costa Luciana S., van Donk Ellen, Hansson Lars‐Anders, Jeppesen Erik, Kruk Carla, Lacerot Gissell, Mazzeo Néstor, De Meester Luc, Moss Brian, Lürling Miquel, Nõges Tiina, Romo Susana, Scheffer Marten (2011). Warmer climates boost cyanobacterial dominance in shallow lakes. Global Change Biology.

[R7] Kovalenko Katya E., Dibble Eric D., Slade Jeremy G. (2010). Community effects of invasive macrophyte control: role of invasive plant abundance and habitat complexity. Journal of Applied Ecology.

[R8] Kuiper Jan J., Verhofstad Michiel J. J. M., Louwers Evelien L. M., Bakker Elisabeth S., Brederveld Robert J., van Gerven Luuk P. A., Janssen Annette B. G., de Klein Jeroen J. M., Mooij Wolf M. (2017). Mowing Submerged Macrophytes in Shallow Lakes with Alternative Stable States: Battling the Good Guys?. Environmental Management.

[R9] Maredová Nela, Altman Jan, Kaštovský Jan (2021). The effects of macrophytes on the growth of bloom-forming cyanobacteria: Systematic review and experiment. Science of The Total Environment.

[R10] Morris Kay, Bailey Paul C. E., Boon Paul I., Hughes Leesa (2006). Effects of plant harvesting and nutrient enrichment on phytoplankton community structure in a shallow urban lake. Hydrobiologia.

[R11] Paerl Hans W., Gardner Wayne S., Havens Karl E., Joyner Alan R., McCarthy Mark J., Newell Silvia E., Qin Boqiang, Scott J. Thad (2016). Mitigating cyanobacterial harmful algal blooms in aquatic ecosystems impacted by climate change and anthropogenic nutrients. Harmful Algae.

[R12] Paxinos R. (2000). A rapid Utermohl method for estimating algal numbers. Journal of Plankton Research.

[R13] Scheffer M., Hosper S.H., Meijer M-L., Moss B., Jeppesen E. (1993). Alternative equilibria in shallow lakes. Trends in Ecology & Evolution.

[R14] Skafi Mourad, Vo Duy Sung, Munoz Gabriel, Dinh Quoc Tuc, Simon Dana F., Juneau Philippe, Sauvé Sébastien (2021). Occurrence of microcystins, anabaenopeptins and other cyanotoxins in fish from a freshwater wildlife reserve impacted by harmful cyanobacterial blooms. Toxicon.

[R15] Trevino-Garrison Ingrid, DeMent Jamie, Ahmed Farah, Haines-Lieber Patricia, Langer Thomas, Ménager Henri, Neff Janet, Van der Merwe Deon, Carney Edward (2015). Human Illnesses and Animal Deaths Associated with Freshwater Harmful Algal Blooms—Kansas. Toxins.

[R16] Zhou Yiwen, Zhou Xiaohong, Han Ruiming, Xu Xiaoguang, Wang Guoxiang, Liu Xiansheng, Bi Fengzhi, Feng Deyou (2017). Reproduction capacity of Potamogeton crispus fragments and its role in water purification and algae inhibition in eutrophic lakes. Science of The Total Environment.

